# Crystal structure of 2,6-bis­(3-hy­droxy-3-methyl­but-1-yn-1-yl)pyridine monohydrate

**DOI:** 10.1107/S2056989020013304

**Published:** 2020-10-09

**Authors:** Take-aki Koizumi, Toshikazu Takata

**Affiliations:** a School of Materials and Chemical Technology, Tokyo Institute of Technology, 4259 Nagatsuta, Midori-ku, Yokohama, 226-8503, Japan

**Keywords:** crystal structure, pyridine, hydrogen bonding, π–π stacking

## Abstract

In the title pyridine derivative, C_15_H_17_NO_2_·H_2_O, the two OH groups are oriented in directions opposite to each other with respect to the plane of the pyridine ring. In the crystal, hydrogen bonds between the pyridine mol­ecule and the water mol­ecule, *viz.* O_hy­droxy_—H⋯O_water_, O_hy­droxy_—H⋯O_hy­droxy_, O_water_—H⋯O_hy­droxy_ and O_water_—H···N_pyridine_, result in the formation of a ribbon-like structure running along [011].

## Abstract 2

In the crystal, the hydrogen bonds between the pyridine mol­ecule and the water mol­ecule, *viz.* O_hy­droxy_—H⋯O_water_, O_hy­droxy_—H⋯O_hy­droxy_, O_water_—H⋯O_hy­droxy_, and O_water_—H⋯N_pyridine_, result in the formation of a ribbon structure running along [01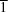].

## Chemical context   

Pyridine derivatives with propargyl alcohol groups as substituents in the 2,6-positions are inter­esting compounds that have been used as synthons of many reactive compounds (Furusho *et al.*, 2004 ▸) and polymers (Miyagawa *et al.*, 2010 ▸, 2011 ▸), as starting materials of helical polymers (Inouye *et al.*, 2004 ▸; Waki *et al.*, 2006 ▸; Abe, Machiguchi *et al.*, 2008 ▸; Abe, Murayama *et al.*, 2008 ▸), and as ligands for transition-metal complexes (Hung *et al.*, 2009 ▸). Since such compounds have rigid structures containing one pyridine nitro­gen and two alcoholic OH groups, they can be used to construct a higher order structure by coordination with metals and/or hydrogen-bond formation at multiple points. The crystal structures of 2,6-bis­(3-methyl­butyn-3-ol)pyridine, **1**, and its complex with tri­phenyl­phosphine oxide (**1**-OPPh_3_) were reported by Holmes *et al.* (2002 ▸). In the crystal of **1**, the mol­ecules form inter­molecular hydrogen bonds with the pyridine ring and the two OH groups; the O—H⋯O hydrogen bonds from a 2_1_ helical chain along the *b-*axis direction. The chains are linked by inter­molecular N⋯H—O hydrogen bonds, forming a layer structure, and then form a stacking structure *via* C—H⋯O inter­actions between the layers. In contrast, in the case of **1**-OPPh_3_, each of the two OH groups forms a hydrogen bond with the O atom of OPPh_3_ without forming a network structure. Hence, it is expected that the crystal packing of **1** strongly depends on the presence or absence of hydrogen bonding. However, to our knowledge, the present examples have only been structurally analysed with 2,6-bis­(propargyl alcohol)-substituted pyridines. In this paper, we report the crystal structure of 2,6-bis­(3-methyl­butyn-3-ol)pyridine monohydrate, **1**·H_2_O.
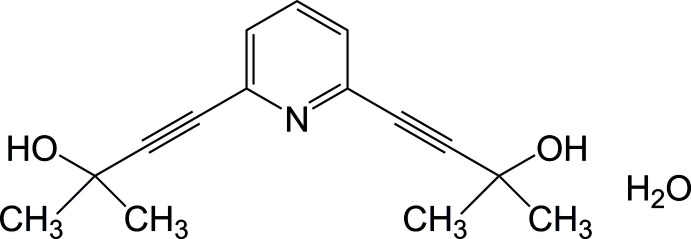


## Structural commentary   

**Figure 1:**
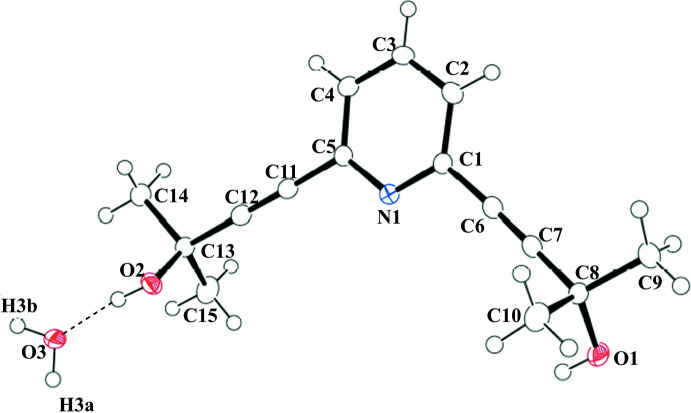
The mol­ecular structure of the title compound with the atom labelling. Displacement ellipsoids are drawn at the 50% probability level. A dashed line indicates the O—H⋯O hydrogen bond.

The mol­ecular structure of the title compound is depicted in Fig. 1 ▸. The bond lengths of two C≡C triple bonds (C6≡C7 and C11≡C12) are 1.199 (2) and 1.191 (2) Å, respectively, consistent with the triple-bond character. The C_*ipso*_—C≡C (C1—C6≡C7 and C5—C11≡C12) and C≡C–C(OH) (C6≡C7—C8 and C11≡C12—C13) bond angles are 176.0 (2), 176.4 (2), 174.6 (2) and 178.5 (2)°, respectively. C6≡C7—C8 is slightly distorted from a linear structure compared to the other bonds. The two OH groups are oriented in directions opposite to each other with respect to the plane of the pyridine ring, and the pyridine ring makes dihedral angles of 50.50 (17) and 57.58 (15)°, respectively, with the C7/C8/O1 and C12/C13/O2 planes.

## Supra­molecular features   

**Figure 2:**
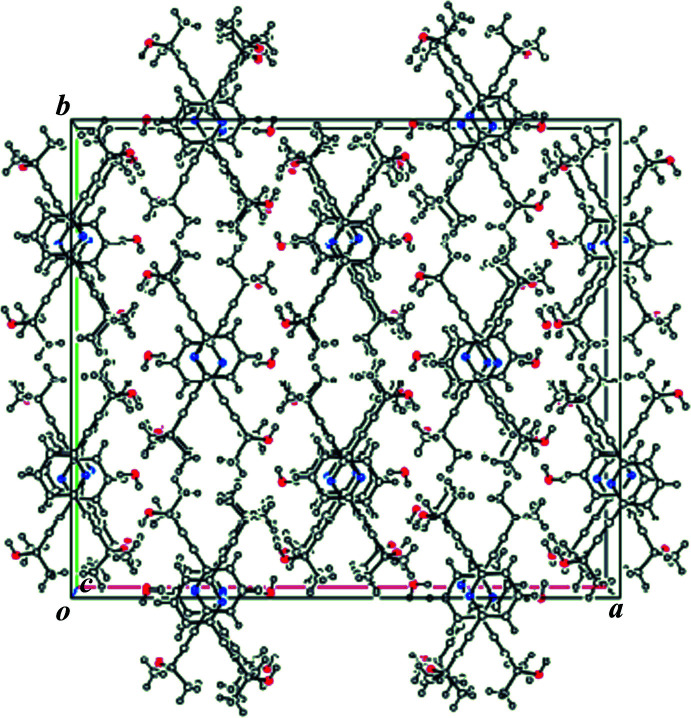
Packing diagram of the title compound, viewed down the *c* axis.

**Figure 3:**
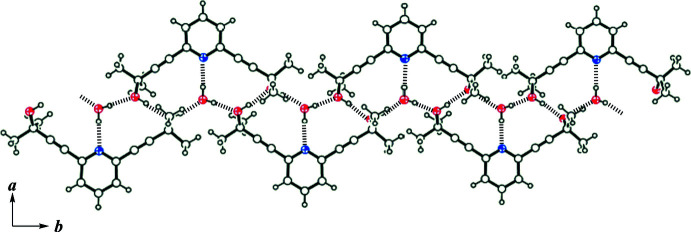
Partial packing diagram of the title compound, showing the O—H⋯O and O—H⋯N hydrogen bonds (dashed lines) between **1** and water mol­ecules.

**Figure 4:**
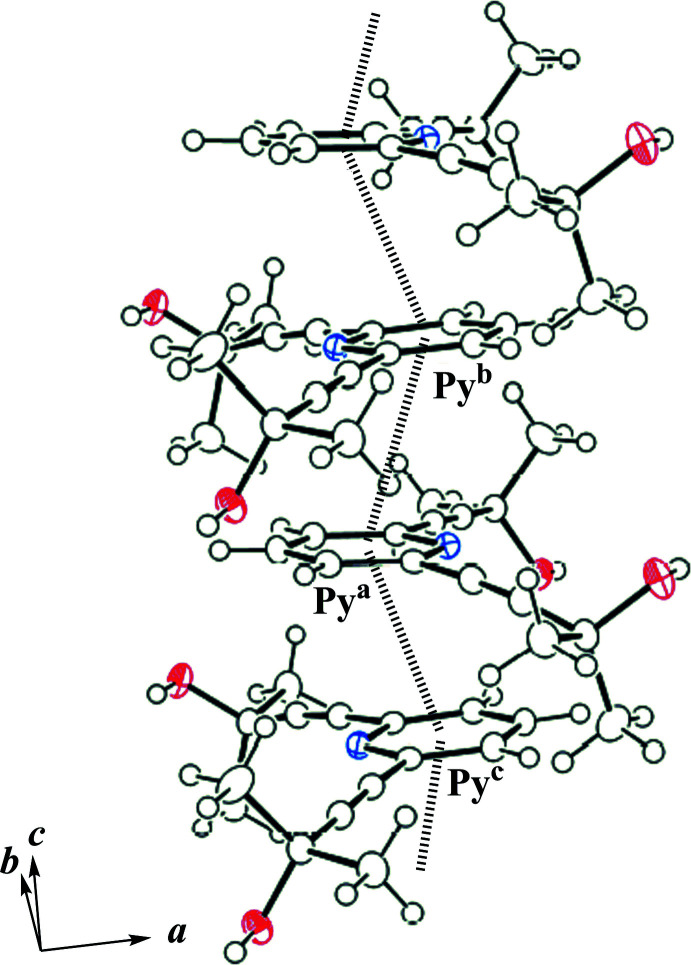
Partial packing diagram of the title compound, showing the chain formation along the *c* axis by π–π inter­actions (dashed lines). [Symmetry codes: (*b*) −*x* + 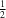, −*y* + 1, *z* + 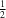; (*c*) −*x* + 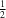, −*y* + 1, *z* − 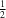.]

Fig. 2 ▸ depicts the packing of **1**·H_2_O along the *c* axis. The water mol­ecules present as the crystallization solvent form inter­molecular O—H⋯O and O—H⋯N inter­actions with the hydroxyl groups and the N atoms of the pyridine unit of mol­ecule **1** (Table 1 ▸), resulting in a ribbon-like structure along [011] (Fig. 3 ▸). The pyridine ring forms π–π stacking inter­actions with that in a neighboring ribbon in an *anti*-parallel mode, resulting in a π–π network along the *c* axis (Fig. 4 ▸). The centroid–centroid distance between the pyridine rings [*Cg*⋯*Cg* ^iv^; symmetry code: (iv) −*x* + 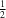, −*y* + 1, *z* + 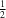] is 3.5538 (11) Å. In the crystal of non-solvated **1** (space group *P*2_1_/*c*; Holmes *et al.*, 2002 ▸), such π–π stacking inter­actions between the pyridine rings are not found.

## Database survey   

The Cambridge Structural Database (CSD version 5.41, update of March 2020; Groom *et al.*, 2016 ▸) has 138 entries for structures containing 2,6-diethynyl­pyridine scaffolds, and for 2,6-bis­(1-propyn-3-ol) derivatives gave two hits. The non-solvated compound 2,6-bis­(3-methyl­butyn-3-ol)pyridine (refcode LUMYEX) and its complex with O=PPh_3_ (LUMYIB) have been reported (Holmes *et al.*, 2002 ▸). The benzene derivative containing two propargyl alcohol units at the 1,3-positions gives 34 hits; however, there is no report of a simple benzene derivative having a structure similar to that of **1**.

## Synthesis and crystallization   

2,6-Bis(3-methyl­butyn-3-ol)pyridine was prepared by using a modified Potts method (Potts *et al.*, 1993 ▸). 2,6-Di­bromo­pyridine (9.1 g, 38 mmol) was reacted with 2-methyl-3-butyn-2-ol (13 g, 151 mmol) using CuI (225 mg, 1.3 mmol)/PdCl_2_(PPh_3_)_2_ (840 mg, 1.3 mmol) as a catalyst in a THF (50 mL)–NEt_3_ (150 mL) solvent for 19 h at room temperature. The resulting dark-brown solution was quenched with an aqueous NH_4_Cl solution and the obtained solid was elimin­ated by celite filtration. The solution was extracted by AcOEt, and the organic phase was dried over MgSO_4_. After filtering off the desiccant, the filtrate was concentrated and subjected to silica-gel chromatography (eluent: AcOEt:hexane 3:2). Single crystals suitable for X-ray diffraction studies were obtained from an ethyl acetate solution *via* slow evaporation in air.

## Refinement   

Crystal data, data collection and refinement details are summarized in Table 2 ▸. Water H atoms and alcohol H atoms were located in a difference-Fourier map, and were refined freely. All of the C-bound H atoms were positioned geometrically (C—H = 0.93 or 0.98 Å), and were refined using a riding model, with *U*_iso_(H) = 1.2*U*_eq_ (aromatic-C) or 1.5*U*_eq_ (methyl-C).

## Supplementary Material

Crystal structure: contains datablock(s) I. DOI: 10.1107/S2056989020013304/is5553sup1.cif

Structure factors: contains datablock(s) I. DOI: 10.1107/S2056989020013304/is5553Isup2.hkl

Supporting information file. DOI: 10.1107/S2056989020013304/is5553Isup3.cml

CCDC references: 2035321, 2035321

Additional supporting information:  crystallographic information; 3D view; checkCIF report

## Figures and Tables

**Table 1:** Hydrogen-bond geometry (Å, °)

*D*—H⋯*A*	*D*—H	H⋯*A*	*D*⋯*A*	*D*—H⋯*A*
O1—H1⋯O2^i^	0.86 (3)	1.90 (3)	2.7640 (15)	175 (3)
O2—H2⋯O3	0.89 (3)	1.82 (2)	2.7052 (17)	170 (3)
O3—H3*A*⋯N1^ii^	0.86 (3)	2.02 (3)	2.8790 (18)	179 (3)
O3—H3*B*⋯O1^iii^	0.83 (3)	2.01 (3)	2.8361 (19)	173 (3)

Symmetry codes: (i) 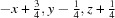; (ii) 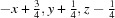; (iii) 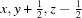.

**Table 2:** Experimental details

Crystal data	
Chemical formula	C_15_H_17_NO_2_·H_2_O
*M*_r_	261.31
Crystal system, space group	Orthorhombic, *F* *d* *d*2
Temperature (K)	113
*a*, *b*, *c* (Å)	31.9834 (14), 27.7358 (13), 6.6610 (4)
*V* (Å^3^)	5908.9 (5)
*Z*	16
Radiation type	Cu *K*α
μ (mm^−1^)	0.66
Crystal size (mm)	0.34 × 0.1 × 0.1

Data collection	
Diffractometer	Rigaku XtaLAB Synergy R, DW system, HyPix
Absorption correction	Multi-scan (*CrysAlis PRO*; Rigaku OD, 2019 ▸)
*T*_min_, *T*_max_	0.817, 1.000
No. of measured, independent and observed [*I* > 2σ(*I*)] reflections	4676, 2071, 2045
*R*_int_	0.015
(sin θ/λ)_max_ (Å^−1^)	0.626

Refinement	
*R*[*F* ^2^ > 2σ(*F* ^2^)], *wR*(*F* ^2^), *S*	0.029, 0.082, 1.04
No. of reflections	2071
No. of parameters	192
No. of restraints	1
H-atom treatment	H atoms treated by a mixture of independent and constrained refinement
Δρ_max_, Δρ_min_ (e Å^−3^)	0.18, −0.20
Absolute structure	Flack *x* determined using 495 quotients [(*I* ^+^)−(*I* ^−^)]/[(*I* ^+^)+(*I* ^−^)] (Parsons *et al.*, 2013 ▸)
Absolute structure parameter	0.02 (11)

Computer programs: *CrysAlis PRO* (Rigaku OD, 2019 ▸), *Olex2.solve* (Bourhis *et al.*, 2015 ▸), *Olex2.refine* (Bourhis *et al.*, 2015 ▸) and *OLEX2* (Dolomanov *et al.*, 2009 ▸).

## supplementary crystallographic information

### Crystal data

**Table:**

C_15_H_17_NO_2_·H_2_O	*D*_x_ = 1.175 Mg m^−^^3^
*M**_r_* = 261.31	Cu *K*α radiation, λ = 1.54184 Å
Orthorhombic, *F**d**d*2	Cell parameters from 4276 reflections
*a* = 31.9834 (14) Å	θ = 4.2–74.9°
*b* = 27.7358 (13) Å	µ = 0.66 mm^−^^1^
*c* = 6.6610 (4) Å	*T* = 113 K
*V* = 5908.9 (5) Å^3^	Plate, white
*Z* = 16	0.34 × 0.1 × 0.1 mm
*F*(000) = 2240	

### Data collection

**Table:**

Rigaku XtaLAB Synergy R, DW system, HyPix diffractometer	2071 independent reflections
Radiation source: Rotating-anode X-ray tube, Rigaku (Cu) X-ray Source	2045 reflections with *I* > 2σ(*I*)
Mirror monochromator	*R*_int_ = 0.015
Detector resolution: 10.0000 pixels mm^-1^	θ_max_ = 75.0°, θ_min_ = 4.2°
ω scans	*h* = −40→25
Absorption correction: multi-scan (CrysAlisPro; Rigaku OD, 2019)	*k* = −22→34
*T*_min_ = 0.817, *T*_max_ = 1.000	*l* = −8→5
4676 measured reflections	

### Refinement

**Table:**

Refinement on *F*^2^	Hydrogen site location: mixed
Least-squares matrix: full	H atoms treated by a mixture of independent and constrained refinement
*R*[*F*^2^ > 2σ(*F*^2^)] = 0.029	*w* = 1/[σ^2^(*F*_o_^2^) + (0.0555*P*)^2^ + 3.7832*P*] where *P* = (*F*_o_^2^ + 2*F*_c_^2^)/3
*wR*(*F*^2^) = 0.082	(Δ/σ)_max_ < 0.001
*S* = 1.04	Δρ_max_ = 0.18 e Å^−^^3^
2071 reflections	Δρ_min_ = −0.19 e Å^−^^3^
192 parameters	Absolute structure: Flack *x* determined using 495 quotients [(*I*^+^)-(*I*^-^)]/[(*I*^+^)+(*I*^-^)] (Parsons *et al.*, 2013)
1 restraint	Absolute structure parameter: 0.02 (11)
Primary atom site location: iterative	

### Special details

**Table:**

Geometry. All esds (except the esd in the dihedral angle between two l.s. planes) are estimated using the full covariance matrix. The cell esds are taken into account individually in the estimation of esds in distances, angles and torsion angles; correlations between esds in cell parameters are only used when they are defined by crystal symmetry. An approximate (isotropic) treatment of cell esds is used for estimating esds involving l.s. planes.

### Fractional atomic coordinates and isotropic or equivalent isotropic displacement parameters (Å^2^)

**Table:**

	*x*	*y*	*z*	*U*_iso_*/*U*_eq_	
O1	0.35948 (3)	0.32551 (4)	0.6275 (2)	0.0246 (3)	
O2	0.34219 (3)	0.65799 (4)	0.3581 (2)	0.0216 (3)	
O3	0.38487 (4)	0.74210 (5)	0.3415 (3)	0.0329 (4)	
N1	0.27513 (4)	0.49371 (5)	0.5965 (3)	0.0166 (3)	
H1	0.3733 (8)	0.3523 (9)	0.623 (5)	0.041 (7)*	
H2	0.3550 (7)	0.6863 (9)	0.339 (5)	0.037 (6)*	
H2A	0.195964	0.419764	0.615414	0.022*	
H3	0.158347	0.492523	0.628127	0.023*	
H3A	0.4117 (8)	0.7423 (8)	0.345 (5)	0.033 (6)*	
H3B	0.3782 (8)	0.7679 (10)	0.288 (5)	0.042 (7)*	
H4	0.194772	0.565882	0.611513	0.022*	
H9A	0.313194	0.255773	0.504956	0.037*	
H9B	0.271146	0.285849	0.469227	0.037*	
H9C	0.290932	0.282373	0.689425	0.037*	
H10A	0.353762	0.360767	0.264577	0.041*	
H10B	0.310243	0.336577	0.202018	0.041*	
H10C	0.350205	0.303410	0.244822	0.041*	
H14A	0.303649	0.733924	0.513951	0.036*	
H14B	0.271251	0.704803	0.651137	0.036*	
H14C	0.269906	0.699027	0.412177	0.036*	
H15A	0.369017	0.641198	0.719381	0.043*	
H15B	0.333112	0.668096	0.843702	0.043*	
H15C	0.364036	0.698483	0.703467	0.043*	
C1	0.25351 (4)	0.45203 (5)	0.6000 (3)	0.0160 (3)	
C2	0.21008 (5)	0.44989 (5)	0.6118 (3)	0.0182 (3)	
C3	0.18798 (5)	0.49280 (6)	0.6182 (3)	0.0193 (4)	
C4	0.20942 (4)	0.53608 (5)	0.6100 (3)	0.0183 (3)	
C5	0.25298 (4)	0.53508 (5)	0.5995 (3)	0.0159 (3)	
C6	0.27810 (5)	0.40863 (6)	0.5837 (3)	0.0186 (4)	
C7	0.29850 (5)	0.37292 (5)	0.5576 (3)	0.0190 (4)	
C8	0.32310 (5)	0.32951 (6)	0.5046 (3)	0.0179 (4)	
C9	0.29729 (5)	0.28434 (6)	0.5457 (3)	0.0244 (4)	
C10	0.33542 (6)	0.33286 (6)	0.2844 (3)	0.0275 (4)	
C11	0.27666 (4)	0.57928 (5)	0.5862 (3)	0.0170 (3)	
C12	0.29570 (4)	0.61579 (6)	0.5643 (3)	0.0178 (3)	
C13	0.31907 (5)	0.66164 (5)	0.5422 (3)	0.0181 (4)	
C14	0.28820 (5)	0.70362 (6)	0.5287 (3)	0.0238 (4)	
C15	0.34897 (6)	0.66791 (7)	0.7178 (4)	0.0290 (4)	

### Atomic displacement parameters (Å^2^)

**Table:**

	*U*^11^	*U*^22^	*U*^33^	*U*^12^	*U*^13^	*U*^23^
O1	0.0193 (5)	0.0161 (5)	0.0383 (9)	−0.0015 (4)	−0.0082 (6)	0.0042 (6)
O2	0.0195 (5)	0.0166 (5)	0.0287 (7)	−0.0014 (4)	0.0053 (6)	0.0013 (6)
O3	0.0154 (5)	0.0246 (6)	0.0586 (11)	−0.0014 (4)	0.0000 (7)	0.0166 (7)
N1	0.0161 (5)	0.0165 (6)	0.0173 (8)	0.0000 (4)	−0.0002 (6)	0.0005 (6)
C1	0.0187 (7)	0.0157 (7)	0.0138 (8)	0.0000 (5)	−0.0010 (7)	0.0008 (7)
C2	0.0186 (6)	0.0176 (7)	0.0182 (9)	−0.0035 (5)	−0.0006 (7)	0.0009 (7)
C3	0.0144 (6)	0.0230 (8)	0.0205 (9)	−0.0004 (6)	−0.0009 (7)	0.0010 (8)
C4	0.0175 (7)	0.0180 (7)	0.0195 (9)	0.0022 (5)	0.0000 (7)	0.0000 (7)
C5	0.0183 (7)	0.0157 (7)	0.0136 (9)	−0.0003 (5)	0.0001 (7)	0.0007 (7)
C6	0.0185 (7)	0.0174 (7)	0.0200 (9)	−0.0027 (5)	−0.0010 (7)	0.0019 (7)
C7	0.0178 (6)	0.0168 (7)	0.0225 (9)	−0.0029 (5)	0.0009 (7)	0.0029 (8)
C8	0.0163 (6)	0.0143 (7)	0.0232 (10)	0.0003 (5)	−0.0017 (7)	0.0024 (7)
C9	0.0228 (7)	0.0164 (7)	0.0339 (11)	−0.0046 (6)	−0.0014 (8)	0.0015 (8)
C10	0.0299 (8)	0.0245 (8)	0.0280 (11)	0.0067 (7)	0.0065 (8)	0.0042 (8)
C11	0.0174 (7)	0.0175 (7)	0.0161 (8)	0.0017 (5)	−0.0005 (7)	−0.0004 (7)
C12	0.0173 (6)	0.0172 (7)	0.0190 (9)	0.0025 (6)	−0.0001 (6)	−0.0009 (7)
C13	0.0179 (7)	0.0135 (7)	0.0229 (10)	0.0004 (5)	0.0002 (7)	0.0001 (7)
C14	0.0230 (7)	0.0161 (7)	0.0322 (11)	0.0031 (6)	0.0025 (8)	0.0023 (8)
C15	0.0340 (9)	0.0203 (8)	0.0327 (11)	−0.0043 (7)	−0.0129 (8)	0.0014 (8)

### Geometric parameters (Å, º)

**Table:**

O2—C13	1.436 (2)	C4—C3	1.384 (2)
O2—H2	0.89 (3)	C13—C14	1.529 (2)
O1—C8	1.427 (2)	C13—C15	1.521 (3)
O1—H1	0.86 (3)	C2—H2A	0.9500
O3—H3A	0.86 (3)	C2—C3	1.385 (2)
O3—H3B	0.83 (3)	C3—H3	0.9500
N1—C1	1.3473 (19)	C9—H9A	0.9800
N1—C5	1.3488 (19)	C9—H9B	0.9800
C1—C6	1.442 (2)	C9—H9C	0.9800
C1—C2	1.393 (2)	C10—H10A	0.9800
C8—C7	1.481 (2)	C10—H10B	0.9800
C8—C9	1.525 (2)	C10—H10C	0.9800
C8—C10	1.522 (3)	C14—H14A	0.9800
C5—C4	1.3952 (19)	C14—H14B	0.9800
C5—C11	1.444 (2)	C14—H14C	0.9800
C12—C11	1.191 (2)	C15—H15A	0.9800
C12—C13	1.482 (2)	C15—H15B	0.9800
C7—C6	1.199 (2)	C15—H15C	0.9800
C4—H4	0.9500		
			
C13—O2—H2	107 (2)	C3—C2—C1	118.31 (13)
C8—O1—H1	109.4 (19)	C3—C2—H2A	120.8
H3A—O3—H3B	105 (2)	C4—C3—C2	119.44 (13)
C1—N1—C5	117.40 (12)	C4—C3—H3	120.3
N1—C1—C6	115.80 (12)	C2—C3—H3	120.3
N1—C1—C2	123.32 (13)	C8—C9—H9A	109.5
C2—C1—C6	120.85 (13)	C8—C9—H9B	109.5
O1—C8—C7	111.09 (14)	C8—C9—H9C	109.5
O1—C8—C9	105.94 (14)	H9A—C9—H9B	109.5
O1—C8—C10	110.27 (13)	H9A—C9—H9C	109.5
C7—C8—C9	109.73 (13)	H9B—C9—H9C	109.5
C7—C8—C10	108.51 (15)	C8—C10—H10A	109.5
C10—C8—C9	111.32 (16)	C8—C10—H10B	109.5
N1—C5—C4	122.83 (14)	C8—C10—H10C	109.5
N1—C5—C11	116.47 (12)	H10A—C10—H10B	109.5
C4—C5—C11	120.68 (14)	H10A—C10—H10C	109.5
C11—C12—C13	178.5 (2)	H10B—C10—H10C	109.5
C6—C7—C8	174.6 (2)	C13—C14—H14A	109.5
C5—C4—H4	120.7	C13—C14—H14B	109.5
C3—C4—C5	118.66 (14)	C13—C14—H14C	109.5
C3—C4—H4	120.7	H14A—C14—H14B	109.5
C12—C11—C5	176.4 (2)	H14A—C14—H14C	109.5
C7—C6—C1	176.0 (2)	H14B—C14—H14C	109.5
O2—C13—C12	106.48 (13)	C13—C15—H15A	109.5
O2—C13—C14	109.61 (14)	C13—C15—H15B	109.5
O2—C13—C15	109.95 (14)	C13—C15—H15C	109.5
C12—C13—C14	109.48 (12)	H15A—C15—H15B	109.5
C12—C13—C15	109.82 (15)	H15A—C15—H15C	109.5
C15—C13—C14	111.38 (14)	H15B—C15—H15C	109.5
C1—C2—H2A	120.8		
			
N1—C1—C2—C3	0.7 (3)	C5—N1—C1—C6	176.16 (16)
N1—C5—C4—C3	0.1 (3)	C5—N1—C1—C2	−1.8 (3)
C1—N1—C5—C4	1.3 (3)	C5—C4—C3—C2	−1.3 (3)
C1—N1—C5—C11	−177.20 (15)	C11—C5—C4—C3	178.62 (17)
C1—C2—C3—C4	0.9 (3)	C6—C1—C2—C3	−177.14 (17)

### Hydrogen-bond geometry (Å, º)

**Table:**

*D*—H···*A*	*D*—H	H···*A*	*D*···*A*	*D*—H···*A*
O1—H1···O2^i^	0.86 (3)	1.90 (3)	2.7640 (15)	175 (3)
O2—H2···O3	0.89 (3)	1.82 (2)	2.7052 (17)	170 (3)
O3—H3*A*···N1^ii^	0.86 (3)	2.02 (3)	2.8790 (18)	179 (3)
O3—H3*B*···O1^iii^	0.83 (3)	2.01 (3)	2.8361 (19)	173 (3)

Symmetry codes: (i) −*x*+3/4, *y*−1/4, *z*+1/4; (ii) −*x*+3/4, *y*+1/4, *z*−1/4; (iii) *x*, *y*+1/2, *z*−1/2.
